# Using a Structural Root System Model to Evaluate and Improve the Accuracy of Root Image Analysis Pipelines

**DOI:** 10.3389/fpls.2017.00447

**Published:** 2017-04-03

**Authors:** Guillaume Lobet, Iko T. Koevoets, Manuel Noll, Patrick E. Meyer, Pierre Tocquin, Loïc Pagès, Claire Périlleux

**Affiliations:** ^1^InBioS-PhytoSYSTEMS, University of LiègeLiège, Belgium; ^2^Institut für Bio-und Geowissenschaften: Agrosphare, Forschungszentrum JülichJülich, Germany; ^3^Plant Cell Biology, Swammerdam Institute for Life Sciences, University of AmsterdamAmsterdam, Netherlands; ^4^INRA, Centre d'Avignon, UR 1115 PSHAvignon, France

**Keywords:** image analysis, root structural model, benchmarking, image library, machine learning

## Abstract

Root system analysis is a complex task, often performed with fully automated image analysis pipelines. However, the outcome is rarely verified by ground-truth data, which might lead to underestimated biases. We have used a root model, ArchiSimple, to create a large and diverse library of ground-truth root system images (10,000). For each image, three levels of noise were created. This library was used to evaluate the accuracy and usefulness of several image descriptors classically used in root image analysis softwares. Our analysis highlighted that the accuracy of the different traits is strongly dependent on the quality of the images and the type, size, and complexity of the root systems analyzed. Our study also demonstrated that machine learning algorithms can be trained on a synthetic library to improve the estimation of several root system traits. Overall, our analysis is a call to caution when using automatic root image analysis tools. If a thorough calibration is not performed on the dataset of interest, unexpected errors might arise, especially for large and complex root images. To facilitate such calibration, both the image library and the different codes used in the study have been made available to the community.

## Introduction

Roots are of utmost importance in the life of plants and hence selection on root systems represents great promise for improving crop tolerance to biotic and abiotic stresses (as reviewed in Koevoets et al., 2016). As such, their quantification is a challenge in many research projects. This quantification is usually two-fold. The first step consists in acquiring images of the root system, either using classic imaging techniques (CCD cameras) or more specialized ones (microCT, X-Ray, fluorescence,…). The next step is to analyse the pictures to extract meaningful descriptors of the root system.

**Figure 1:**
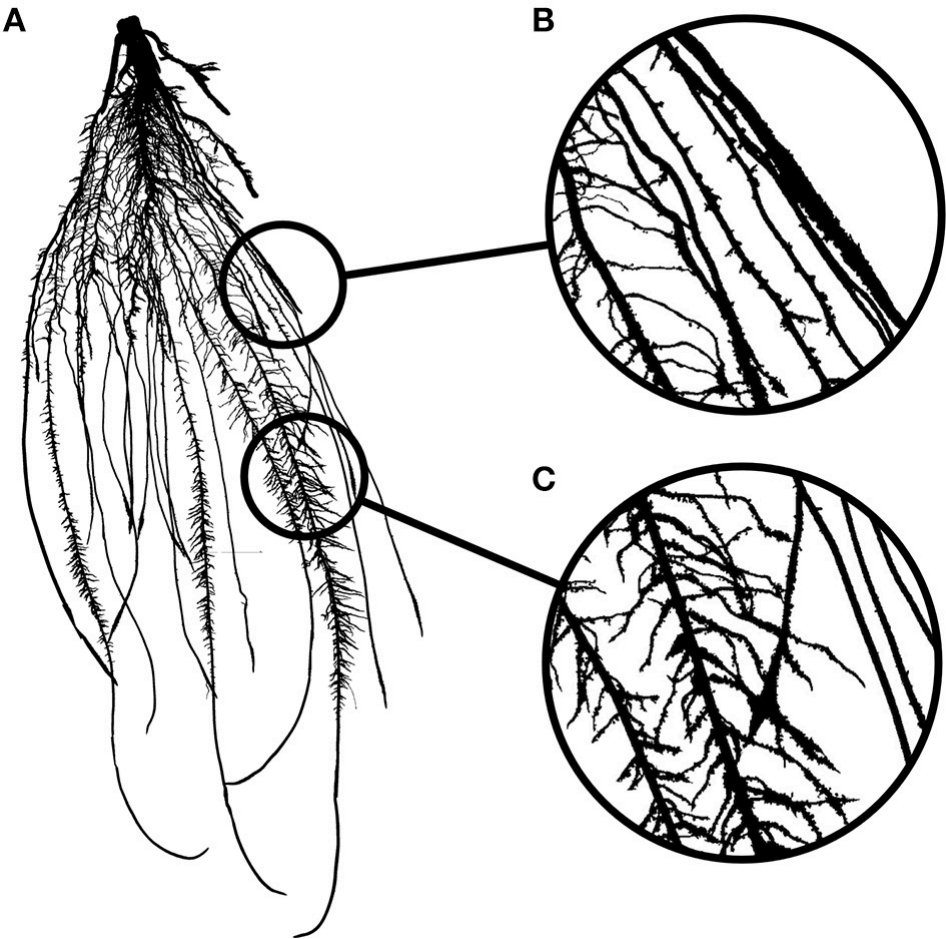
**(A)** Image of a 2-week old maize root system grown in rhizotron. **(B)** Close-up showing overlapping roots. **(C)** Close-up showing crossing roots.

To paraphrase the famous Belgian surrealist painter, René Magritte: “Figure 1A is not a root system.” Figure 1A is an image of a root system and that distinction is important. An image is indeed a two-dimensional representation of an object, which is usually three-dimensional. Nowadays, measurements are generally not performed on the root systems themselves, but on the images, and this raises some issues.

Image analysis is the acquisition of traits (or descriptors) describing the objects contained in a particular image. In a perfect situation, these descriptors would accurately represent the biological object of the image with negligible deviation from the biological truth (or data). However, in many cases, artifacts might be present in the images so that the representation of the biological object is not accurate anymore. These artifacts might be due to the conditions under which the images were taken or to the object itself. Mature root systems, for instance, are complex branched structures, composed of thousands of overlapping (Figure 1B), and crossing segments (Figure 1C). These features are likely to impede image analysis and create a gap between the descriptors and the data.

Root image descriptors can be separated into two main categories: morphological and geometrical descriptors. Morphological descriptors refer to the shape of the different root segments forming the root system (Table 1). They include, among others, the length and diameter of the different roots. For complex root system images, morphological descriptors are difficult to obtain and are prone to error as mentioned above. Geometrical descriptors give the position of the different root segments in space. They summarize the shape of the root system as a whole. The simplest geometrical descriptors are the width and depth of the root system. Since these descriptors are mostly defined by the external envelope of the root system, crossing and overlapping segments have little impact on their estimation and hence they can be considered as relatively errorless. Geometrical descriptors are expected to be loosely linked to the actual root system topology, since identical shapes could be obtained from different root systems (the opposite is true as well). They are usually used in genetic studies, to identify genetic bases of root system shape and soil exploration.

**Table 1:** **Root system parameters used as ground-truth data**.

**Name**	**Description**	**Unit**
tot_root_length	The cumulative length of all roots	mm
tot_1_order_length	The cumulative length of all root axes	mm
tot_2+_order_length	The cumulative length of all lateral roots	mm
mean_1_order_length	The mean first-order roots length	mm
mean_2+_order_length	The mean lateral root length	mm
n_1_orders	The total number of first order roots	–
n_2+_orders	The total number of lateral roots	–
mean_2+_order_density	The mean lateral root density: for each first-order root, the number of lateral roots divided by the axis length (total length).	mm-1
mean_1_order_diam	The mean diameter of the first-order roots	mm
mean_2+_order_diam	The mean diameter of the lateral roots	mm
mean_2+_order_angle	The mean insertion angle of the lateral roots	°

Several automated analysis tools were designed in the last few years to extract both types of descriptors from root images (Armengaud et al., 2009; Galkovskyi et al., 2012; Pierret et al., 2013; Bucksch et al., 2014). However, the validation of such tools is often incomplete and/or error prone. For technical reasons, the validation is usually performed on a small number of ground-truth images of young root systems. In agreement, most analysis tools are specifically designed for this kind of root systems. In the few cases where validation is performed on large and complex root systems, it is usually not on ground-truth images, but in comparison with previously published tools (measurement of X with tool A compared with the same measurement with tool B). This might seem a reasonable approach, regarding the scarcity of ground-truth images of large root systems. However, the inherent limitations of these tools, such as scale or root system type (fibrous- vs. tap-roots) are often not known. Users might not even be aware that such limitations exist and apply the provided algorithm without further validation on their own images. This can lead to unexpected errors in the final measurements.

One strategy to address the lack of in-depth validation of image analysis pipelines would be to use synthetic images generated by structural root models (models designed to recreate the physical structure and shape of root systems). Many structural root models have been developed, either to model specific plant species (Pagès et al., 1989), or to be generic (Pagès et al., 2004, 2013). These models have been repeatedly shown to faithfully represent the root system structure (Pagès and Pellerin, 1996). In addition, they can provide the ground-truth data for each synthetic root system generated, independently of its complexity. However, they have not been used for validation of image analysis tools (Rellán-Álvarez et al., 2015), with one exception performed on young seedling unbranched roots (Benoit et al., 2014).

Here we (i) illustrate the use of a structural root model, Archisimple, to systematically analyse and evaluate an image analysis pipeline and (ii) use the model-generated images to improve the estimation of root traits.

## Materials and methods

### Nomenclature used in the paper

**Ground-truth data**: The real (geometrical and morphometrical) properties of the root system as a biological object. They are determined by either manual tracking of roots or by using the output of simulated root systems.
**(Image) Descriptor**: Property of the root image. It does not necessarily have a biological meaning.
**Root axes:** First order roots, directly attached to the shoot.
**Lateral roots:** Second- (or lower) order roots, attached to another root.

### Creation of a root system library

**Figure 2:**
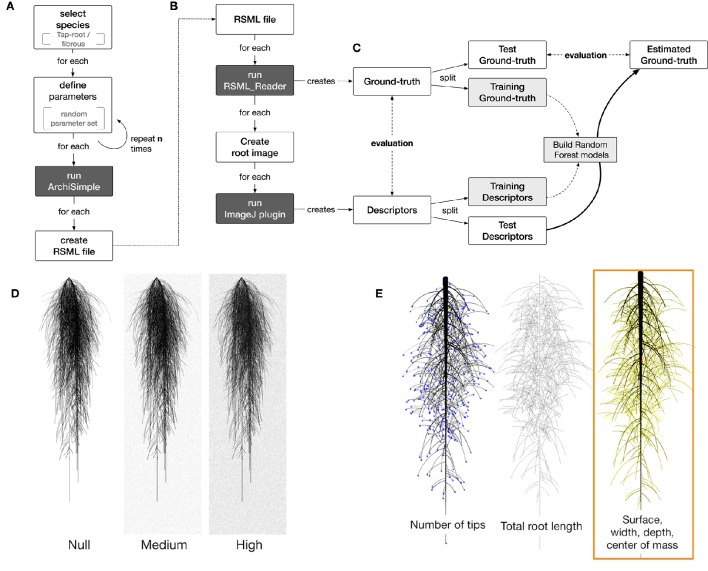
**Overview of the workflow used in this study**. **(A)** Generation of root systems using Archisimple. **(B)** Creation and analysis of root images. **(C)** Use of Random Forest algorithms to better estimate root system ground-truths. **(D)** Illustration of the different noise levels used in the analysis. **(E)** Example of descriptors extracted with RIA-J.

We used the model ArchiSimple, which was shown to allow the generation of a large diversity of root systems with a minimal amount of parameters (Pagès et al., 2013). To produce a large library of root systems, we ran the model 10,000 times, each time with a random set of parameters (Figure 2A). For each simulation, the growth and development of the root system were constrained in two dimensions.

The simulations were divided into two main groups: fibrous and tap-rooted. For the fibrous simulations, the model generated a random number of root axes and secondary (radial) growth was disabled. For tap-root simulations, only one root axis was produced and secondary growth was enabled (the extent of which was determined by a random parameter).

The root system created in each simulation was stored in a Root System Markup Language (RSML) file. Each RSML file was then read by the RSML Reader plugin from ImageJ to extract ground-truth data for the library (Lobet et al., 2015). These ground-truth data included geometrical and morphological parameters (Table 1). For each RSML data file, the RSML Reader plugin also created three JPEG images (at a resolution of 300 DPI) for each root system. To simulate one type of image degradation, we added different levels of noise to the images (using the Salt and Pepper Filter in ImageJ) (Figure 2D). For each root system, we computed overlapping index as the number of root segments having an overlap with other root segments over the total number of root segments.

### Root image analysis

Each generated image was analyzed using a custom-made ImageJ plugin, Root Image Analysis-J (or RIA-J). For each image, we extracted a set of classical root image descriptors, such as the total root length, the projected area, and the number of visible root tips (Figure 2E). In addition, we included shape descriptors such as the convex-hull area or the exploration ratio (see Supplemental File 1 for details of RIA-J). The list of traits and algorithms used by our pipeline is listed in Table 2. Distribution of the different descriptors is given in the Supplemental Figure 2.

**Table 2:** **Root image descriptors extracted by RIA-J**.

**Name**	**Description**	**Unit**	**Reference**
**MORPHOLOGY**			
area	Projected area of the root system	mm^2^	Galkovskyi et al., 2012
length	Length of the skeleton of the root system image	mm	Galkovskyi et al., 2012
tip_count	Number of end branches in the root system skeleton	–	
diam_mean	Mean diameter of the root object in the image	mm	
**GEOMETRY**			
width	The maximal width of the root system	mm	–
depth	The maximal depth of the root system	mm	–
width_depth_ratio	Ratio between the width and the depth of the root system	–	Galkovskyi et al., 2012
com_x–com_y	Relative coordinates of the center of mass of the root system	–	Galkovskyi et al., 2012
convexhull	Area of the smallest convex shape encapsulating the root system	mm^2^	Galkovskyi et al., 2012
exploration	Ratio between the convex hull area and the projected area	–	Galkovskyi et al., 2012

### Data analysis

Data analysis was performed in R (R Core Team)^1^. Plots were created using *ggplot2* (Wickham, 2009) and *lattice* (Sarkar, 2008).

The Mean Relative Errors (MRE) were estimated using the equation:

$$\begin{array}{l} MRE=\frac{\sum_{1}^{n}\frac{\left|{\bar{y}}_{i}\;-\;y_{i}\right|}{{\bar{y}}_{i}}}{n} \end{array}$$

where *n* is the number of observations, ${\bar{y}}_{i}$ is the ground-truth and *y*_*i*_ is the estimated ground-truth.

### Random forest framework

A *random forest* is a state-of-the-art machine learning algorithm typically used for making new predictions (in both classification and regression tasks). Random Forests can perform non-linear predictions and, thus, often outperform linear models. Since its introduction by Breiman (2001), Random Forests have been widely used in many fields from gene regulatory network inference to generic image classification (Huynh-Thu et al., 2013; Marée et al., 2016). Random Forest relies on growing a multitude of decision trees, a prediction algorithm that has shown good performances by itself but, when combined with other decision trees (hence the name forest), returns predictions that are much more robust to outliers and noisy data (see bootstrap aggregating, Breiman, 1996).

In a machine learning setting one is given a set *D* = {(*x*_1_, *y*_1_), (*x*_2_, *y*_2_), …, (*x*_*n*_, *y*_*n*_)},

where $x_{i}=\left(x_{i}^{1},x_{i}^{2}\ldots ,x_{i}^{s}\right)$ is an element of a *s*−dimensional *feature space X*,

and $y_{i}=\left(y_{i}^{1},y_{i}^{2},\ldots ,y_{i}^{t}\right)$ an element of a *t*−dimensional *response space Y*.

The learning task is to find a model

$$\begin{array}{l} M:X\to Y \end{array}$$

that predicts the data in a good way, where goodness is measured with regard to an error function *L*.

A decision tree *T*_*D*_ is a machine learning method that, for a dataset *D*, constructs a binary tree with each node representing a binary question and each leaf a value of the response space. In other words, a prediction can be made from an input value by looking at the set of binary questions that leads to a leaf (e.g., is the first-order root bigger than q1 and if yes is the number of second-order roots smaller than q2 and if no, …).

Each decision is based upon exactly one feature and is used for deciding which branch of the tree a given input value must take. Hence a decision tree splits successively the set *D* into smaller subsets and assigns them a value *y*_*i*_ = *T*_*D*_(*x*_*i*_) of the response space.

The choice of the feature used for splitting depends on a relevance criterion. In our setting, the default relevance criterion from the randomForest R package (CRAN randomForest, 2015), namely the Gini index, has been used.

A Random Forest

(1) $$\begin{array}{r} F_{D}={\left(T_{D,k}\right)}_{k\;\in \;I}\;\text{where}\;I\;=\left\{1,2,\ldots .l\right\} \end{array}$$

consists of *l* decision trees *T*_*D, k*_, where several key parameters such as the feature space, are chosen randomly (hence the word Random in the algorithm name). While using a random subspace strongly accelerates the growth of a single tree, it can also decrease its accuracy. However, the use of large number of trees counterbalance advantageously those two effects. The final prediction for each input value *x*_*i*_ corresponds to the majority vote of all the decision trees of the forest *T*_*D, k*_(*x*_*i*_) in a classification setting while an average of all predicted values is used in a regression task.

#### Framework description

Our method consists of three typical steps:

1. A preprocessing step, where we replace missing values of the training set.
2. A model generation step where, for each response variable, we generate different models according to two Random Forest parameters (number of trees and number of splits).
3. A model selection step, where we choose the best performing pair of parameters of the previous step for each one of the response variable.

#### Preprocessing

Missing values in our dataset might arise due to highly noisy images, where the measurement of certain descriptors has been infeasible. To deal with this issue, we first replaced missing values.

This is done using the imputation function of the randomForest R package. It replaces all missing values of a response variable by the median and then a Random Forest is applied on the completed data to predict a more accurate value. We favored 10 trees for computing the new value over the default value of 300 as we found that it offered sufficiently accurate results for our application while being much faster.

#### Model generation

In the model generation step, for each of the response variables, several forests with different number of trees and different number of splits (*t*_*i*_, *m*_*j*_) are tested. In practice, the training set *D*_*train*_ is divided into *m*_*j*_ disjunct subsets $D_{train}^{m_{j}}$ and on each of those, a Random Forest $F_{D_{train}^{m_{j}}}$ is trained on a growing number of *t*_*i*_ random trees.

#### Model selection

Given a new data point *x*, each model predicts a response variable *y* by averaging the predicted values $F_{D_{train}^{m}}\left(x\right)$, i.e.,

$$\begin{array}{l} ŷ\;=\;M_{D_{train}}^{t,m}\left(x\right)=\frac{1}{m}\overset{m}{\underset{k\;=\;1}{\sum }}F_{D_{train}^{k}}\left(x\right) \end{array}$$

Then in a final step an estimate of the root-mean-square (RMSE) generalized error on the test set ***D***_***t**est*_ is computed, where RSME is defined as

$$\begin{array}{l} RMSE\;=\;\sum_{i=1}^{n}\sqrt{{\left(y_{i}-{\bar{y}}_{i}\right)}^{2}} \end{array}$$

for ***D***_***t**est*_={(x_1_,y_1_),(x_2_,y_2_),…,(x_n_,y_n_)}.

Finally, the model with the parameter pair (*t*,*m*) having the minimal error (on the separate test set) is chosen in order to make the predictions.

### Data availability

All data used in this paper (including the image and RSML libraries) are available at the address http://doi.org/10.5281/zenodo.208214

An archived version of the codes used in this paper is available at the address http://doi.org/10.5281/zenodo.208499

An archived version of the machine learning framework is available at the address https://github.com/FaustFrankenstein/RandomForestFramework/releases/tag/v1.0

## Results and discussions

### Production of a large library of ground-truth root system images

We combined existing tools into a single pipeline to produce a large library of ground-truth root system images. The pipeline combines a root model (ArchiSimple, Pagès et al., 2013), the Root System Markup Language (RSML) and the RSML Reader plugin from ImageJ (Lobet et al., 2015). In short, ArchiSimple was used to create a large number of root systems, based on random input parameter sets. Each output was stored as an RSML file (Figure 2A), which was then used by the RSML Reader plugin to create a graphical representation of the root system (as a. jpeg file) and a ground-truth dataset (Figure 2B). Details about the different steps are presented in the Materials and Methods section.

We used the pipeline to create a library of 10,000 root system images, separated into fibrous (multiple first order roots and no secondary growth) and tap-root systems (one first order root and secondary growth). The ranges of the different ground-truth data are shown in Table 3 and their distribution is shown in the Supplemental Figure 1.

**Table 3:** **Ranges of the different ground-truth data from the root systems generated using ArchiSimple**.

**Variable**	**Minimum value**	**Maximum value**	**Unit**
**FIBROUS**			
tot_root_length	110	73971	mm
width	0.76	302	mm
depth	50	505	mm
n_1_order	1	20	–
tot_1_order_length	79	5409	mm
mean_1_order_length	27	470	mm
mean_1_order_diameter	0.2	0.4	mm
mean_2+_order_density	0	5	root/mm
n_2+_orders	0	4448	–
tot_2+_order_length	0	71556	mm
mean_2+_order_length	0	50	mm
mean_2+_order_diameter	0	0.3	mm
mean_2+_order_angle	0	88	°
**TAP ROOTED**			
tot_root_length	78	41870	mm
width	0.1	173	mm
depth	55	505	mm
n_1_order	1	1	–
tot_1_order_length	59	509	mm
mean_1_order_length	59	509	mm
mean_1_order_diameter	0.2	16	mm
mean_2+_order_density	0	2.3	root/mm
n_2+_orders	0	3353	–
tot_2+_order_length	0	40225	mm
mean_2+_order_length	0	51	mm
mean_2+_order_diameter	0	2.2	mm
mean_2+_order_angle	0	97	°

**Figure 3:**
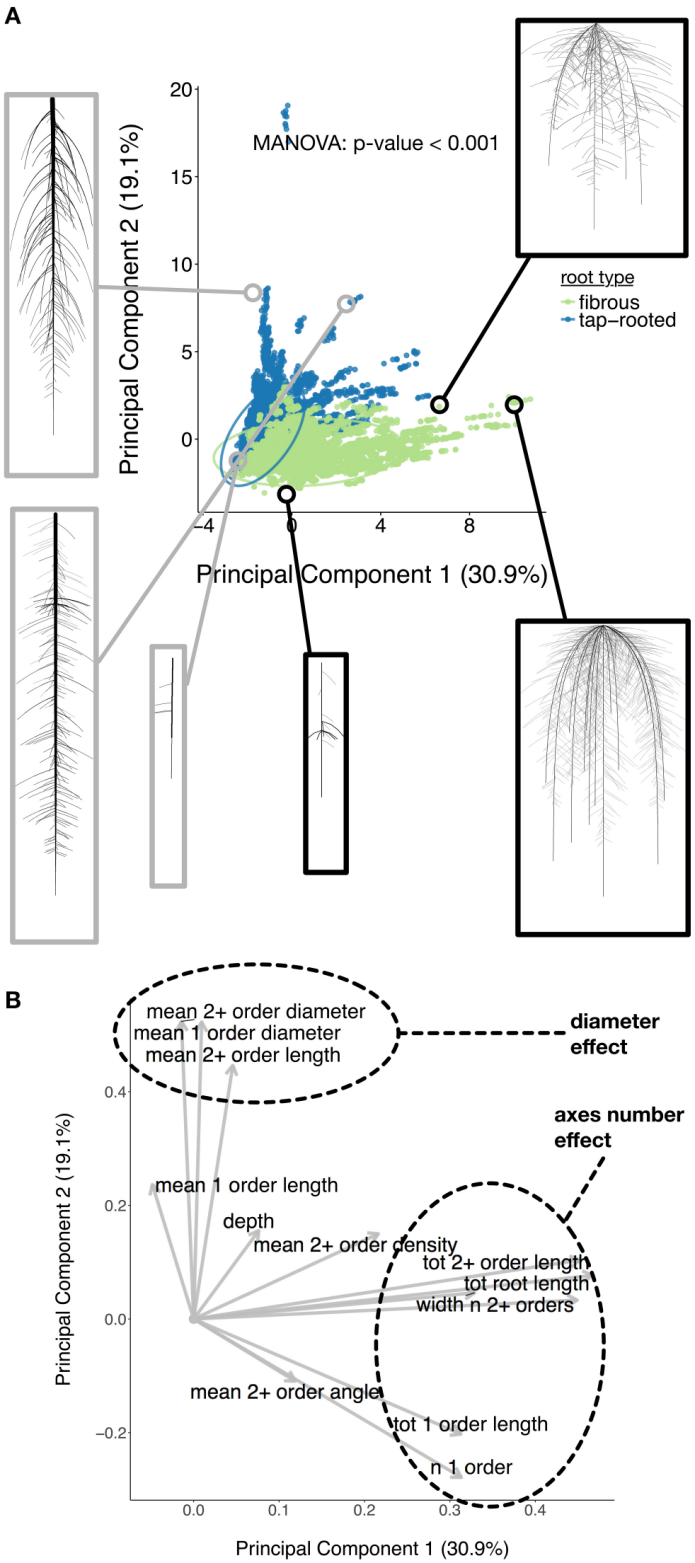
**(A)** Principal Component Analysis of the root ground-truth dataset. Images of the selected root systems have been added for illustration. **(B)** Loadings of the Principal Component Analysis.

We started by evaluating whether fibrous and tap-root systems should be separated during the analysis. We performed a Principal Component Analysis on the ground-truth dataset to reduce its dimensionality and assess if the *type* grouping influenced the overall dataset structure (Figure 3A). Fibrous and tap-root systems formed distinct groups (MANOVA *p* < 0.001), with limited overlap. The first principal component, which represented 30.9% of the variation within the dataset, was mostly influenced by the number of first-order axes. The second principal component (19.1% of the variation) was influenced, in part, by the root diameters. These two effects were consistent with the clear root system type grouping, since they expressed the main difference between the two groups of root-system *types*. Therefore, since the *type* grouping had such a strong effect on the overall structure, we decided to separate them for the following analyses.

To demonstrate the utility of a synthetic library of ground-truth root systems, we analyzed every image of the library using a custom-built root image analysis tool, RIA-J. We decided to do so since our purpose was to test the usefulness of the synthetic analysis and not to assess the accuracy of existing tools. Nonetheless, RIA-J was designed using known and published algorithms, often used in root system quantification. A detailed description of RIA-J can be found in the Materials and Methods section and Supplemental File 1.

**Figure 4:**
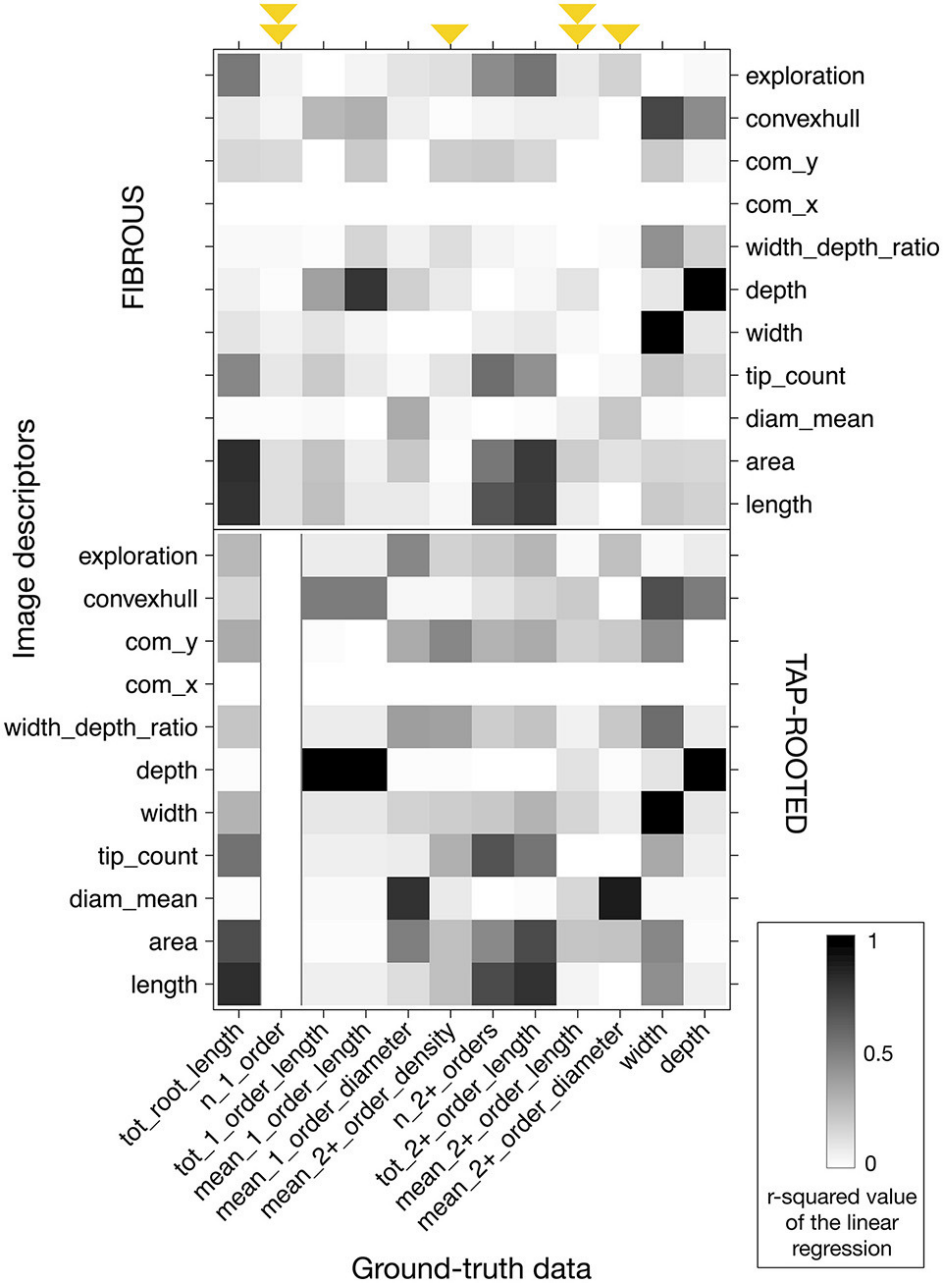
**Heatmap of the r-squared values between the different image descriptors and the ground-truth values, for the images without any noise**. Black represents an r-squared value of 1; white represents a value of 0. Upper panel: fibrous root dataset. Lower panel: tap-root dataset. Arrows highlight the ground-truth data that cannot be accurately described with the different descriptors. The arrows were doubled when it was the case for both fibrous and tap-rooted root systems.

We extracted 10 descriptors from each root system image (Table 2) and compared them with the ground-truth data. For each pair of descriptor-data, we performed a linear regression and computed its r-squared value. Different types of information are highlighted in Figure 4. First, using a ground-truth image library allows for a quick and systematic analysis of all the descriptors extracted by the image analysis pipeline. Second, it allows researchers to identify which traits can be accurately evaluated (or not) and by which descriptors. Third, for some ground-truth data, such as the mean length of second order roots or the number of first order roots, it shows that none of the classical descriptors gave a good estimation (Figure 4, highlighted with arrows). Finally, the figure highlights that some correlations were different for fibrous- and tap-root systems. As an example, the correlation found between the *mean_2*+*_order_diameter* and *diam_mean* estimators was better for fibrous roots than within the tap-root dataset. Consequently, validation of the different image analysis algorithms should be performed, at least, for each group. An algorithm giving good results for a fibrous root system might fail when applied to tap-rooted ones.

### Errors from image descriptors are likely to be non-linear across root system sizes and image qualities

**Figure 5:**
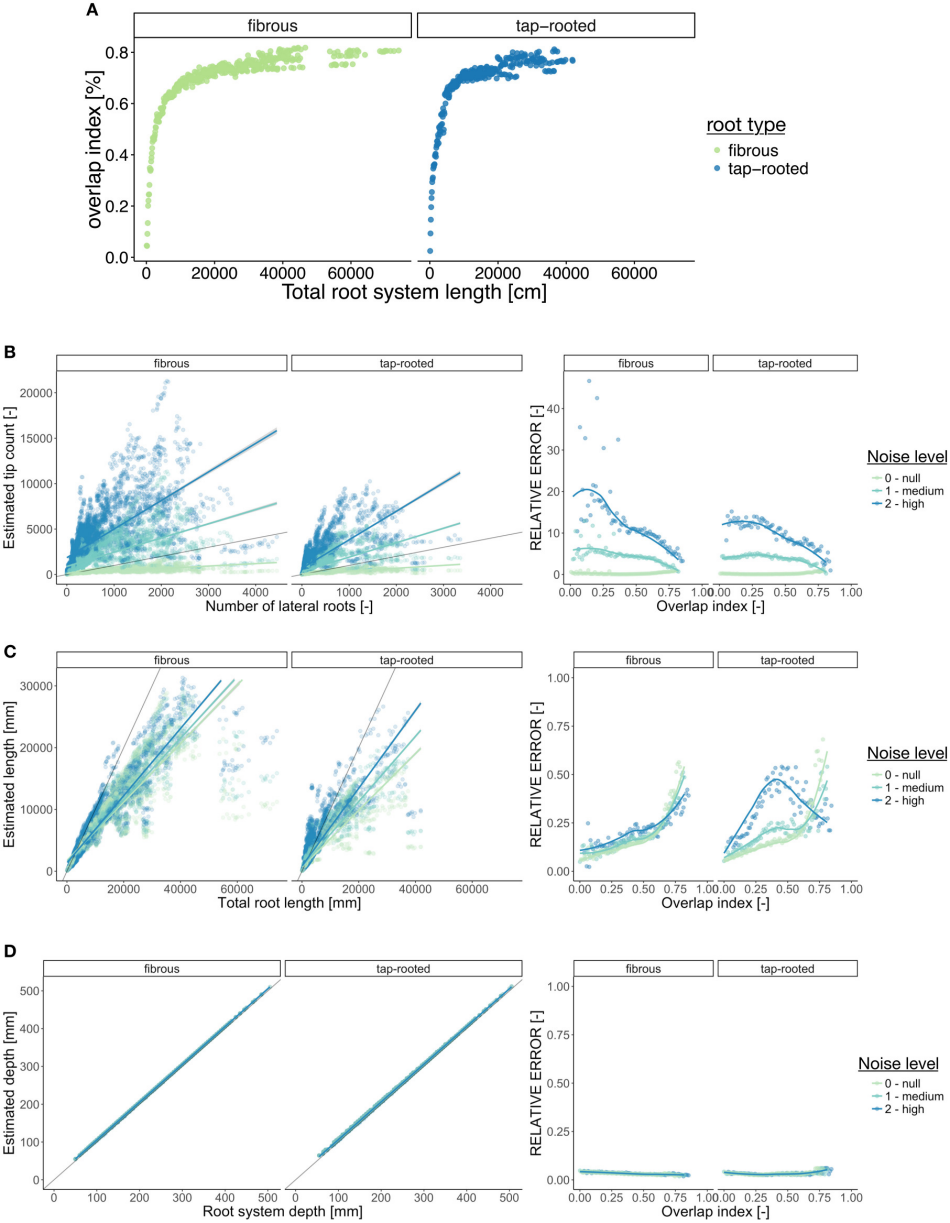
**Error estimation for three ground-truth parameters**. **(A)** Evolution of the overlap index (proportion of root overlapping) with the root system size. **(B–D)** Left panel shows the relationship between the descriptors and the corresponding ground-truth variables. Right panels show the evolution of the Mean Relative Error (MRE) as a function of the overlap index. For the MRE calculations, the continuous variables were discretized in groups. **(B)** Number of lateral roots. **(C)** Total root length. **(D)** Root system depth. In the left panels, the gray line indicates the diagonal (1:1 relation).

In addition to being related to the species of study, estimation errors are likely to increase with the root system size. As the root system grows and develops, the number of crossing and overlapping segments increases (Figure 5A), making the subsequent image analysis potentially more difficult and prone to error. However, a systematic analysis of such error is seldom performed.

Estimation errors are also likely to increase as the image quality decreases. Here we artificially added one type of noise (random “salt and pepper” particles) to the images, with two intensity levels. It should be noted that virtually any type of image degradation could be added to the original images using custom image filters (e.g., using ImageJ). Different types of degradation are expected to generate different levels of estimation errors.

Figure 5 shows the relationship between the ground-truth and descriptor values for three parameters: the total root length (Figure 5B), the number of roots (Figure 5C), and the root system depth (Figure 5D). For each of these variables, we quantified the Mean Relative Error (see Materials and Methods for details) as a function of the overlap index. This was done for three levels of noise added to the images (“null,” “medium,” and “high”). We can observe that for the estimation of both the total root length and the number of lateral roots, the Mean Relative Error increased with the size of the root system (Figures 5B–C). As stated above, such increase of the error was somehow expected with increasing complexity. Moreover, depending on the metric of interest, such as the number of root tips, low image quality can result in high level of error. For other traits, such as the root system depth, no errors were expected (*depth* is supposedly an error-less variable) and the Mean Relative Error was close to 0 whatever the size of the root system and image quality.

The results presented here are tightly dependent on the specific algorithms used for image analysis and hence might be different for other published tools. However, they are a call for caution when analyzing root images: unexpected errors in ground-truth estimation can arise. Our image library can be used to better identify the errors generated by other analysis tools, current or future.

### Roadmap for root image analysis tools calibration

To improve the calibration and validation of future root image analysis tools, we propose the following procedure:

1. Develop the new root image analysis pipeline;
2. Use it to analyse the images from the synthetic root library described here;
3. Compare the results from the new analysis with the corresponding ground-truth;
4. Identify, and clearly state, the type of root systems for which the pipeline works accurately;
5. When releasing the new pipeline, inform the users about the possible errors identified.

### Using the synthetic library to train machine learning algorithms

The main advantage of creating a synthetic library is to generate paired datasets of image descriptors and their corresponding ground-truth values. Having information on both can, in theory, be used to either calibrate the image analysis pipeline or to identify the best descriptors for the ground-truth traits of interest. Here, we explored the second approach and used a Random Forest algorithm to find which combination of descriptors would best describe each ground-truth data (see Material and Methods for details). In short, we randomly divided the whole dataset into training (3/4) and testing subsets (1/4). The training set was used to create a Random Forest model for each ground-truth data, which was then we applied to the test set. The accuracy of these new predictions was then compared to the accuracy of the direct method (single descriptors) (Figure 2C).

**Figure 6:**
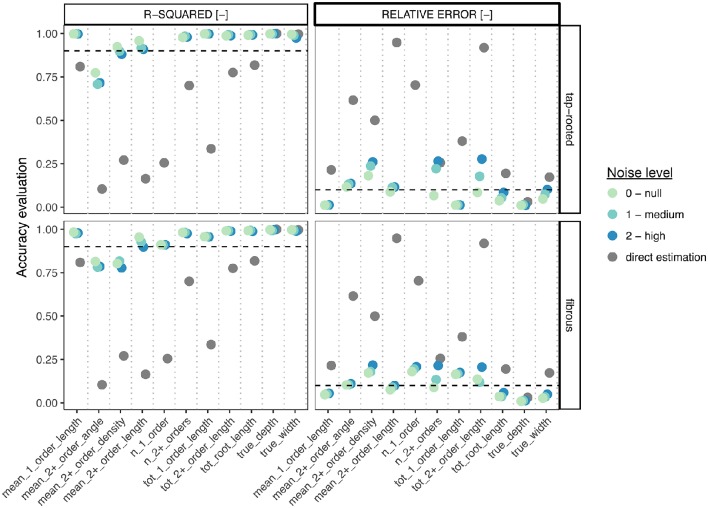
**Comparison between the direct trait and the Random Forest approach, for the different root system types and the different levels of noise**. For each metric, we computed both the r-squared value from the linear regression between the estimation and the ground-truth (left panels), as well as the Mean Relative Error (right panel). The gray points represent the values obtained with the direct estimation (best descriptor, no noise). Color points represent the values obtained with the Random Forest approach, for different levels of noise. The dotted lines show the 0.9 (r-squared) and 0.1(MRE) thresholds.

Figure 6 shows the comparison of the accuracy (both the r-squared values from linear regressions and the Mean Relative Error, MRE) of both methods for each ground-truth data. We can clearly see that the Random Forest approach performed always better (sometimes substantially) than the direct approach, even for images with high level of noise. In addition, for most traits, the r-squared and MRE values were above 0.9 and below 0.1 respectively, which is very good, especially for such a wide range of images. In addition, the Random Forest approach allowed the correct estimation of traits that were difficult to estimate with the direct approach (such as the number of first-order axes or the mean second-order root density).

**Figure 7:**
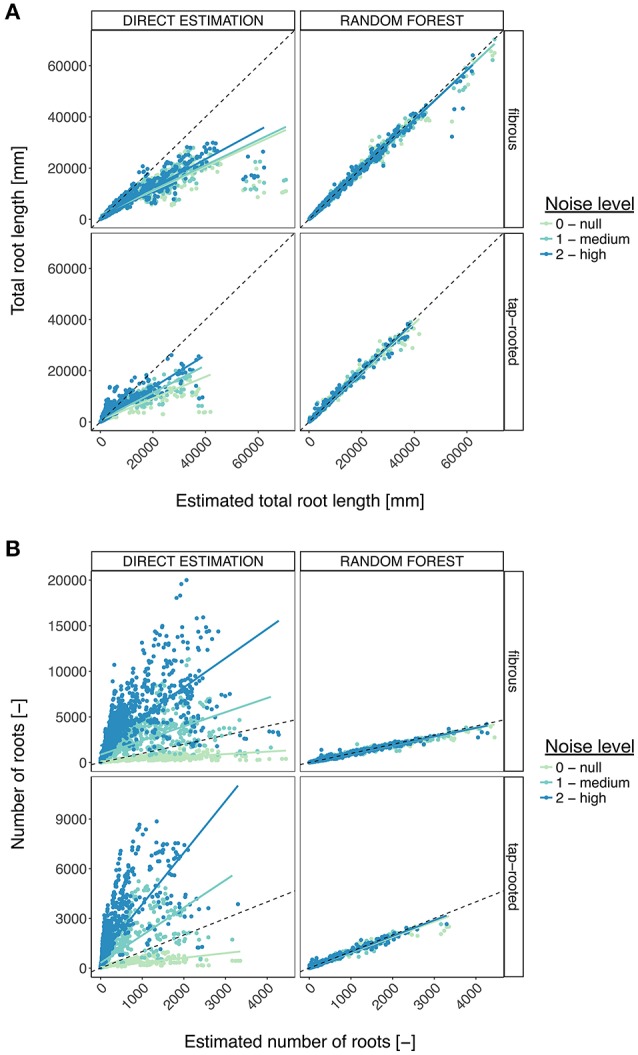
**Comparison between the direct trait estimation and the Random Forest approach, for the different root system types and the different levels of noise**. **(A)** Comparison, for the total root length, of the accuracy of both approaches. The dotted line represents the diagonal. The plain line represents the linear regression. **(B)** Same, for the number of roots.

Figure 7 shows the detailed comparison of both methods for the estimation of the total root length. Again, a clear improvement was visible with the Random Forest method, leading to small errors, even with large root systems and noisy images.

Here we presented how machine learning algorithms (Random Forest), could be used in combination with a synthetic image library to improve the estimation of root system traits. Although both the training and test datasets used were made of synthetic images, we believe this approach presents an interesting perspective for the analysis of experimental images.

Indeed, a root architectural model can be used to build a custom library of synthetic images from a set of parameters evaluated on a small number of plants from the experimental dataset. Such library could then be used to train the machine learning model which, in turn, will enable the automatic evaluation of root traits from the remaining experimental images. Alternatively, the algorithm could be directly trained on a subset of experimental data obtained by manual or semi-automatic analyses to be then automatically applied to the rest of the dataset. One must keep in mind that the output of the machine learning strongly depends upon the quality of the dataset used for its training and hence must be analyzed carefully.

## Conclusions

The automated analysis of root system images is routinely performed in many research projects. Here we used a library of 10,000 synthetic images to estimate the accuracy and usefulness of different image descriptors extracted with a homemade root image analysis pipeline. Our study highlighted some limitations and biases of the image analysis process.

We found that the type of root system (fibrous vs. tap-rooted), its size and complexity, as well as the quality of the images had a strong influence on the accuracy of some commonly used image descriptors and their meaning and relevance for ground-truth extraction. So far, a large proportion of the root research has been focused on seedlings with small root systems and has *de facto* avoided such errors.

However, as the research questions are likely to focus more on mature root systems in the future, these limitations will become critical. We showed that synthetic datasets can be used for calibration or modeling (machine learning) steps that allow ground-truth extraction from comparable images. We then hope that our library will be helpful for the root research community to evaluate and improve other image analysis pipelines.

## Author contributions

GL, LP, PT, IK, MN, and CP designed the study. IK developed the image analysis pipeline RIA-J. MN and PM developed the Random Forest framework. GL generated the image library and did the data analysis. LP developed the Archisimple model. All authors have participated in the writing of the manuscript.

## Funding

This research was funded by the Interuniversity Attraction Poles Programme initiated by the Belgian Science Policy Office, P7/29. GL and MN are grateful to the F.R.S.-FNRS for a postdoctoral research grant (1.B.237.15F) and doctoral grant (1.A.320.16F), respectively.

### Conflict of interest statement

The authors declare that the research was conducted in the absence of any commercial or financial relationships that could be construed as a potential conflict of interest.

## Footnotes

^1^R Core Team R: A Language and Environment for Statistical Computing.

## Supplementary material

Supplemental Figure 1 **Distribution of the properties of the modeled root images**.

Supplemental Figure 2 **Distribution of the descriptors of the modeled root images**.

Supplemental File 1 **Definitions of the different descriptors extracted by RIA-J**.
