# Supplementary figures and images for: Using a Structural Root System Model to Evaluate and Improve the Accuracy of Root Image Analysis Pipelines
Source: Front Plant Sci. 2017 Apr 3;8:447. doi: 10.3389/fpls.2017.00447 (PMC5376626; doi:10.3389/fpls.2017.00447)

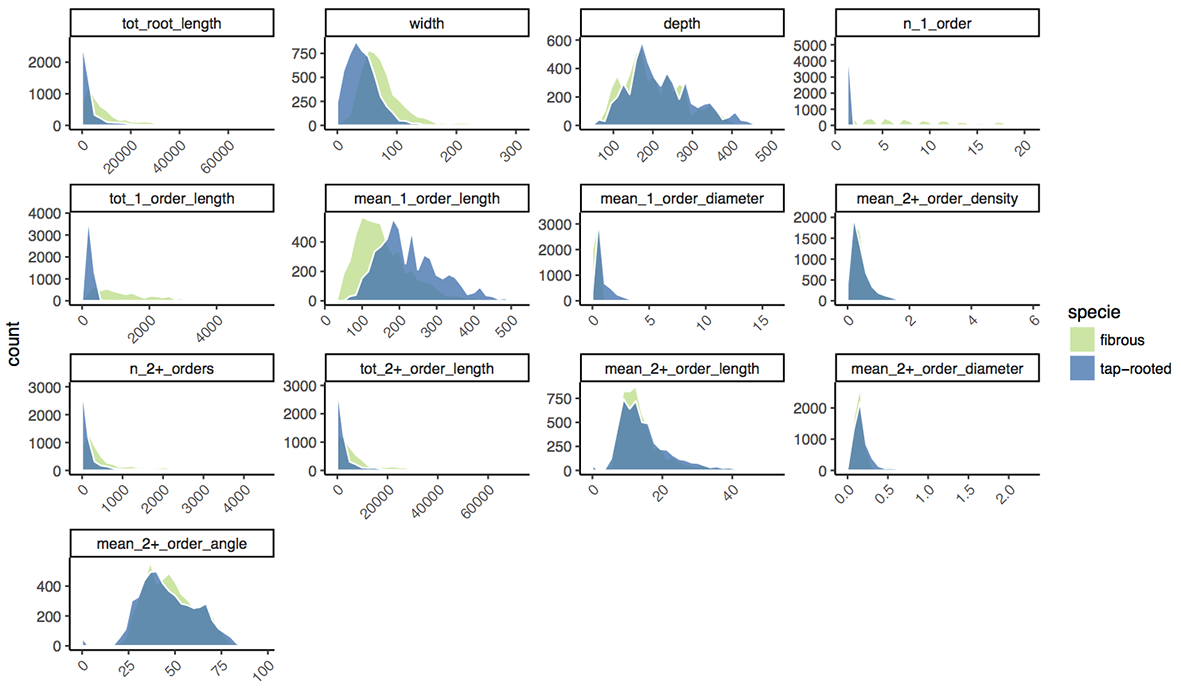

Supplement: Supplemental Figure 1 — Distribution of the properties of the modeled root images. [file Image1.TIFF]

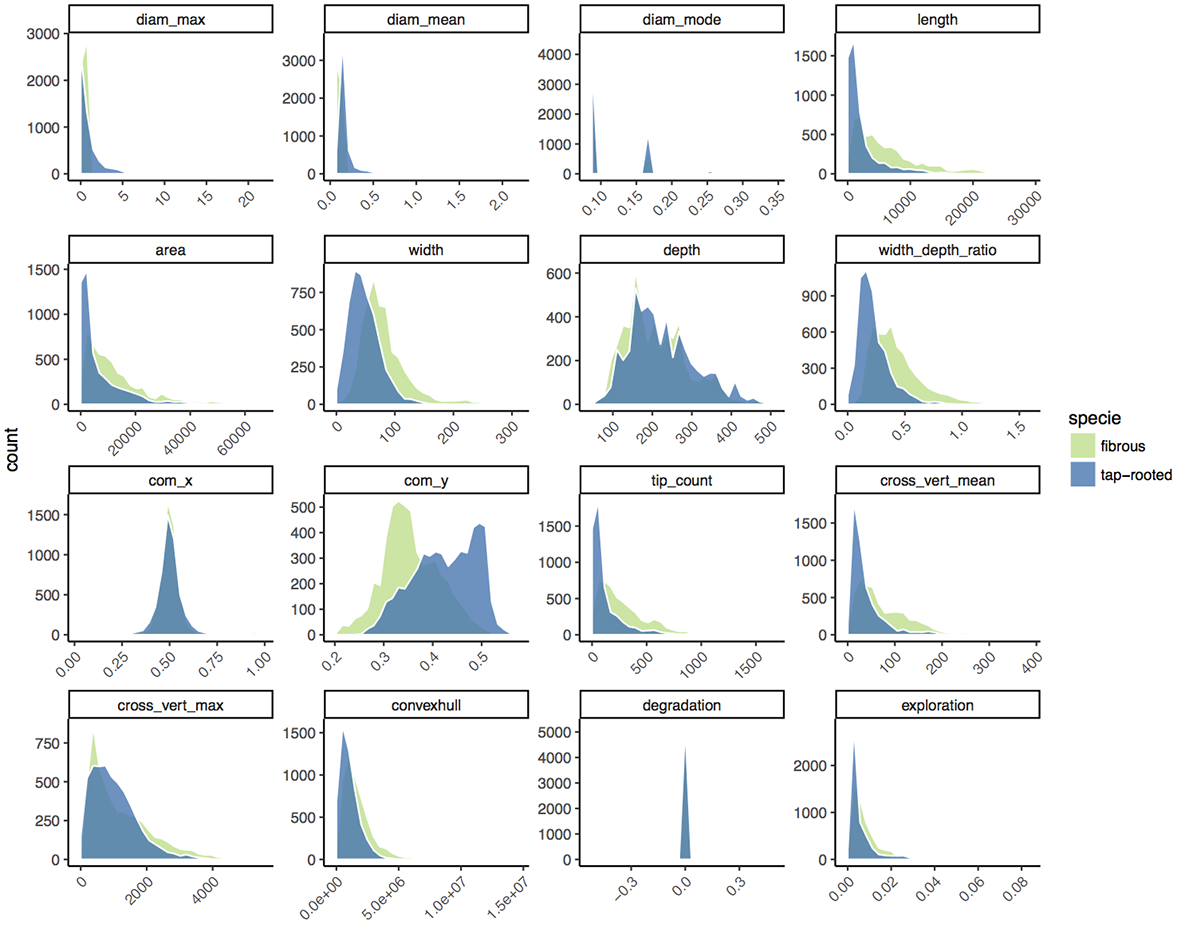

Supplement: Supplemental Figure 2 — Distribution of the descriptors of the modeled root images. [file Image2.TIFF]
