# Supplementary material for: Using a Structural Root System Model to Evaluate and Improve the Accuracy of Root Image Analysis Pipelines
Source: Front Plant Sci. 2017 Apr 3;8:447. doi: 10.3389/fpls.2017.00447 (PMC5376626; doi:10.3389/fpls.2017.00447)
Supplement: Supplemental File 1 — Definitions of the different descriptors extracted by RIA-J. [file DataSheet1.PDF]

Using a structural root system model to evaluate and improve the accuracy of image analysis pipelines

G Lobet, I Koevoets, M Noll, P Tocquin, PE Meyer, L Pagès and C Périlleux

Supplemental file 1:
Definition of the metrics used in RIAJ

The image analysis pipeline was developed as an ImageJ plugin. Its first step is to threshold the image, using the Otsu algorithm. Once the image was thresholded, we extract the following metrics:

Area: the number of black pixel in the image

Length: the number of pixels in the skeleton of the root system. The skeleton is obtained by repeatedly removing the black pixels at the edge of the object, such as only one pixel is remaining. We use the native function in ImageJ for the skeleton generation

Tip Count: The number of end-points in the skeleton image. And end-point was defined as having only one black pixel neighbor.

Mean diameter: To compute the diameter, we computed a distance map from the thresholded image, such as each pixel has the value of its distance to the closest with pixel. The distance map was computed using the native function in ImageJ (Process>Binary>Distance Map).Then, we combine this distance map image with the skeleton (logical AND operation). The resulting image is a skeleton of the root system, were the value of each pixel is the local diameter. The mean diameter was computed as the mean of all these pixels.

Width / depth ratio (fig. 1.A) is probably the simplest shape descriptor, indicating the relative investment of the root system in depth compare to the horizontal exploration of the soil.

The **Center of Mass** (fig. 1.A) is the weighted average of the x and y coordinates all pixels of the root system. It reflects the relative distribution of roots within the root system. A root system with many root in its upper layers will have a Center of Mass with a low Y value (closer to the top).

The **Exploration** ratio is the ratio between the **convex hull area** (fig. 1.B) and the projected area of the root system (the number of root pixels in the root system image). The ratio represents how dense the root system is, relative its total soil volume exploration.

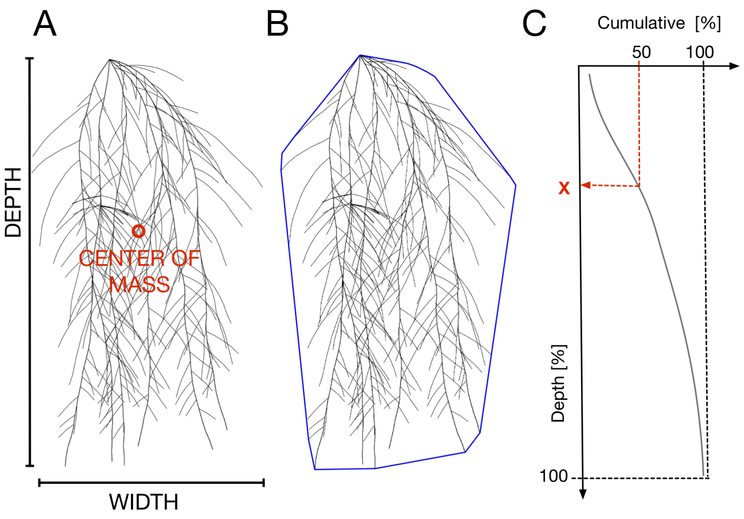

Figure 1: Shape descriptors. A. Width, depth and center of mass. B. Convex hull. C. Depth at 50% of cumulative variable (see text for explanation).

Finally, accumulation variables (**width50** and **count50**) are indicators of the width / root number distribution along the depth profile (Bucksch *et al.*, 2014). For each variable, we compute its cumulative value along the depth profile. From the final cumulative value, we find the relative depth at with 50% of this value is reached.

References

Bucksch A, Burridge J, York LM, Das A, Nord E, Weitz JS, Lynch JP. 2014. Image-based high-throughput field phenotyping of crop roots.
